# Paratransgenesis: a promising new strategy for mosquito vector control

**DOI:** 10.1186/s13071-015-0959-2

**Published:** 2015-06-24

**Authors:** André Barretto Bruno Wilke, Mauro Toledo Marrelli

**Affiliations:** Departamento de Epidemiologia, Faculdade de Saúde Pública, Universidade de São Paulo, Av. Dr. Arnaldo 715, São Paulo, SP CEP-01246-904 Brazil

**Keywords:** Paratransgenesis, Vector control, Bacteria, Transgenic mosquito, Neglected diseases

## Abstract

The three main mosquito genera, *Anopheles*, *Aedes* and *Culex*, transmit respectively malaria, dengue and lymphatic filariasis. Current mosquito control strategies have proved unsuccessful, and there still is a substantial number of morbidity and mortality from these diseases. Genetic control methods have now arisen as promising alternative strategies, based on two approaches: the replacement of a vector population by disease-refractory mosquitoes and the release of mosquitoes carrying a lethal gene to suppress target populations. However, substantial hurdles and limitations need to be overcome if these methods are to be used successfully, the most significant being that a transgenic mosquito strain is required for every target species, making genetically modified mosquito strategies inviable when there are multiple vector mosquitoes in the same area. Genetically modified bacteria capable of colonizing a wide range of mosquito species may be a solution to this problem and another option for the control of these diseases. In the paratransgenic approach, symbiotic bacteria are genetically modified and reintroduced in mosquitoes, where they express effector molecules. For this approach to be used in practice, however, requires a better understanding of mosquito microbiota and that symbiotic bacteria and effector molecules be identified. Paratransgenesis could prove very useful in mosquito species that are inherently difficult to transform or in sibling species complexes. In this approach, a genetic modified bacteria can act by: (a) causing pathogenic effects in the host; (b) interfering with the host’s reproduction; (c) reducing the vector’s competence; and (d) interfering with oogenesis and embryogenesis. It is a much more flexible and adaptable approach than the use of genetically modified mosquitoes because effector molecules and symbiotic bacteria can be replaced if they do not achieve the desired result. Paratransgenesis may therefore become an important integrated pest management tool for mosquito control.

## Review

### Mosquito-borne diseases

Since the late nineteenth century, when mosquitoes were first associated with the transmission of pathogens to humans and other vertebrates, a number of mosquito species have been intensively studied. The three main mosquito genera, *Anopheles*, *Aedes* and *Culex*, transmit the causative agents of malaria, dengue and filariasis, respectively, as well as a variety of mosquito-borne zoonotic arboviruses, such as West Nile Virus (WNV), Saint Louis Encephalitis Virus (SLEV) and Eastern Equine Encephalitis Virus (EEE). Because of the inherent difficulty of mosquito control and therefore the implantation of surveillance strategies, there still is a significant morbidity and mortality due to these diseases [1–4], along with an increase in various other diseases caused by insect-vectored pathogens of epidemiological importance [2, 3, 5–8]. The increase in the geographic distribution of mosquitoes is followed by the emergence of viruses and diseases in new areas [9].

For example, the number of malaria cases has increased as a result of deteriorating health systems, increased anopheline resistance to insecticides, *Plasmodium* resistance to antimalarial drugs and the time taken to develop an effective vaccine. Malaria is the world’s most epidemiologically important vector-borne disease according to World Health Organization [1]. Cases of the disease have been reported in more than 100 countries, and approximately 3 billion people around the world live in endemic areas. There are estimated to be over 200 million cases of malaria and 800 thousand malaria-related deaths every year [1, 10, 11].

Dengue, including dengue hemorrhagic fever and dengue shock syndrome (DSS), which is transmitted by *Aedes* mosquitoes, is rapidly becoming a worldwide disease, threatening a third of the world’s population. There are estimated to be 50 to 100 million cases every year [2, 3, 5, 6, 8, 12]. *Aedes aegypti* is the main vector of the dengue virus and is a highly invasive species that has been inadvertently spread around the world by man [2, 3]. It is found in tropical and subtropical regions, mainly in urban environments, where it has adapted to artificial breeding sites. Bed nets are ineffective, since *Ae.aegypti* has its peak activity during the day, making breeding-site management and the use of insecticides the main forms of control [13].

Lymphatic filariasis, which is caused by *Wuchereria bancrofti*, affects more than 120 million people around the world [4, 14]. It is transmitted mainly by mosquitoes of the genus *Culex* and causes irreversible damage to the host [11, 15]. A considerable number of arboviruses can also be transmitted by many species in the genus *Culex*, some of which are of great public health importance, such as WNV [16].

Although great efforts have been made, there are currently no effective vaccines against dengue, filariasis or malaria, and specific treatments are only available for malaria and filariasis. Therefore, the most effective way to prevent vector-borne diseases is to control mosquito populations [17–21]. This review, which was motivated by the situation described above, discusses the current state of knowledge about paratransgenesis in mosquitoes, emphasizing the failure of different mosquito control strategies and alternative paratransgenic approaches.

### Mosquito-control strategies

Vector-control strategies in the last century were based on chemical agents such as dichloro-diphenyl-trichloroethane (DDT). Although insecticides have been successfully used to control mosquitoes of the genera *Aedes* and *Anopheles*, current ecological and environmental protection standards do not allow such approaches because of the adverse effects of many insecticides on non-target species, including humans, their environmental impact, the contamination of soil and water and the development of selective processes and subsequent mosquito resistance to insecticides [5].

New strategies therefore had to be created to replace the use of insecticides. These include Integrated Pest Management (IPM), in which insecticides should be used only as a last resort in epidemics. IPM guidelines are based on environmental planning, public awareness and biological control and seek to control the mosquito population more efficiently while preserving the environment from contamination [22].

The great reproductive capacity and high genomic flexibility of mosquitoes make management of these insects very difficult. Their high genomic flexibility is demonstrated by the resistance of mosquito populations to chemical and biological insecticides as well as by their ability to adapt to different environmental conditions and to climate changes [7, 18, 23–25]. Therefore, effective alternative forms of control that can be used on a large scale and are environmentally friendly are urgently needed and should be included in IPM [26–28].

Genetic control methods have proven to be promising strategies for mosquito control. Two such methods have been developed: the use of refractory mosquitoes to replace vector populations and the release of mosquitoes carrying a lethal gene to suppress target populations. The first approach is self-sustaining, and only one or a few releases are needed for the exogenous gene to increase its frequency and be fixed in the target population (inoculative releases). The second strategy is self-limiting, since repeated releases are necessary to keep the lethal gene acting in the target population (inundative releases) [29–31].

Genetic control strategies for population replacement rely on inhibiting the development of pathogens at some point in the vector life cycle. When released into the area of interest, the transgene is introduced in the target population, blocking transmission of the disease caused by that particular pathogen [32]. This technique is still under development and has not yet been used on a large scale. Self-sustaining techniques rely basically on two components: a refractoriness mechanism and a gene driver to spread the target gene in the population. Several mosquito species have been genetically modified to be resistant and disrupt parasites transmission cycles [33–35]. The classic study by Ito et al. [33] transformed *Anopheles stephensi* to express a small peptide, known as salivary gland and midgut peptide 1 (SM1), in the midgut under the control of the carboxypeptidase promoter, blocking around 80 % of oocyst development. Since this study, several species of anophelines have been transformed to express anti-malaria molecules [36–39], including *Anopheles gambiae*, the main malaria vector in Africa [38], with a similar blocking effect. However, to effectively control vectored diseases such as malaria, it is crucial to establish a repertoire of effector genes and tissue-specific promoters for their expression to enable these genes to have maximum effect, i.e., make the mosquito 100 % refractory to the parasite with minimum fitness cost. There are still several epidemiological risks and major problems that need to be dealt with before population-replacement approaches can be tested in the field [39].

The Release of Insects with Dominant Lethality (RIDL) system was proposed simultaneously by Heinrich & Scott [40] and Thomas et al. [41] and is based on the integration of a dominant lethal gene associated with a female-specific promoter. When released into the wild, genetically modified males will mate with wild females, resulting in viable male offspring but inviable female offspring because of the gene’s sex-specific lethality, which is expressed only in the absence of a chemical repressor used to silence the effector gene [42]. A variety of genes are available for this purpose, the only requirement being that they cause death when expressed [43]. When a female-specific promoter is used, males carry the gene but do not express it and are able to survive with or without the chemical repressor, while females die in the absence of the repressor [44].

As suppression strategies based on genetic modification have begun to be used in the field, the lack of regulations governing the use of GMM has become a significant challenge. This lack of regulations is not restricted to developing countries and strategies based on the use of recombinant DNA. Laws and regulations need to be created, risk analyses carried out and public opinion assessed, a process made all the more arduous by the regulatory agencies’ lack of experience. Furthermore, the best strategy for implementing control programs based on genetic modification is neither simple nor obvious [11]. The self-limiting nature of reproductively sterile insects (whether sterilized by radiation, cytoplasmic incompatibility or RIDL) tends to simplify the use of this technique in the field, since the transgene is not fixed in the wild population [11].

From the perspective of transgenic insect-release programs, such as RIDL, it is extremely important to detect any resistance in the target population that could adversely affect the effectiveness of the program soon enough to take measures to remedy the problem. Monitoring strategies must provide suitable warning about these problems in time to allow them to be remedied [11].

### Transgenic mosquitoes or paratransgenesis in mosquitoes?

Considerable effort has been devoted to the genetic transformation of mosquitoes, and although refractory mosquitoes and mosquitoes carrying a lethal gene have been produced [33, 44], there are still significant issues to be dealt with before the use of these techniques in the field can be considered, such as drive mechanisms, transposon stability issues, sibling species complexes and multiple subspecies of mosquitoes [33, 45]. All of these factors need to be taken into account before the introduction of transformed mosquitoes into wild populations can be considered [46]. Although these obstacles can be overcome with time, the issue of new, much-needed control measures remains. In this regard, paratransgenesis (the genetic modification of symbiotic organisms, such as bacteria and fungi) would appear to be the most suitable solution [31, 46–48].

### The paratransgenesis alternative

Paratransgenesis is based on the use of symbiotic bacteria to express effector molecules inside the target vector [47–51]. The symbiotic bacteria are genetically modified to express effector molecules and then reintroduced into the mosquito, where they produce the desired effect [49, 52–55]. Although studies of the microbiota of wild mosquitoes have been carried out, identifying its full spectrum and potential is yet to be done [14, 44, 52, 56–61]. Knowledge of mosquito microbiota is essential for a paratransgenic system to work, and is particularly important to identify bacteria that are well established in mosquitoes and can be transmitted to the next generation [14].

For a bacteria to be used in paratransgenesis three key components are required: an effector molecule that achieves the desired effect; a mechanism to display or excrete the effector molecule on the surface of the bacteria; and bacteria that can survive in the mosquito long enough to produce the expected amount of effector molecules and thereby achieve the desired effect in the mosquito [45, 46].

Identification of suitable commensal bacteria that are non-pathogenic to humans or animals among the many organisms that insects harbor, particularly in their digestive systems, is paramount for the success of a paratransgenic system [62]. In mosquitoes these bacteria are involved in various biological functions associated with digestion, primarily in the midgut. There is a close association between blood-dependent insects and symbiotic microorganisms that help the anabolic processes of vitellogenesis and ovogenesis [63]. Eradication of these bacteria leads to a decline in fecundity and a slower growth rate. Interference with the digestion of proteins in mosquito blood meals can reduce fecundity and may represent a new approach for controlling mosquito populations and preventing the transmission of pathogens [63].

The chosen bacteria should be capable of colonizing a wide variety of mosquito species so that they can be deployed in different species and isolated strains. Producing recombinant bacteria in sufficient numbers is much simpler than creating transgenic mosquitoes [46, 64].

Furthermore, the number of bacteria increases dramatically (100 to 1000 of times) after ingestion of blood [65], resulting in a proportional increase in the amount of effector molecules expressed and secreted by GM bacteria [66], leading to various possible outcomes: obstructing pathogen transmission, reducing the mosquito’s vector capacity, preventing fertilization of eggs, interfering with embryogenesis and causing the death of the mosquito [49, 52, 54, 55].

### Symbiotic bacteria and fungi for use in paratransgenesis

Symbiotic bacteria can be found in several insects, such as in *Ae. aegypti* where they probably play a critical role in metabolic processes and may be important for these insects as they colonize their internal organs and other tissues. The elimination of obligatory symbionts would result in fitness loss. Wide-ranging studies of these symbiotic microorganisms might provide important data for the development of new control strategies [63].

Pidiyar et al. [14] found a wide range of symbiotic bacteria in various mosquito species: *Bacillus spp*., *Staphylococcus spp*., *Pseudomonas spp*. and *Aeromonas culicicola* in *Culex quinquefasciatus*; *Serratia marcescens* and *Klebsiella ozonae*; *Pseudomonas aeruginosa* and *Enterobacter agglomerans* in *Culex pipiens*; and two strains of *A. culicicola* in *Ae. aegypti*. Dinparast et al. [67] reported thirteen species of bacteria in *An. Maculipennis* and *An. Stephensi*.

In studies with malaria vectors, a wide variety of bacteria species were isolated from the midguts of wild mosquitoes of the genus *Anopheles* in East and West Africa [53]. In southern Mexico, Gonzalez-Ceron et al. [57] found *Enterobacter amnigenus*, *Enterobacter cloacae*, *Enterobacter sp*., *S. marcescens*, and *Serratia sp*. in *Anopheles albimanus*.

Bacteria of the genus *Asaia* have a stable association and are capable of quickly colonizing tissues in several mosquito species, such as *An. gambiae*, *An. stephensi*, *Aedes albopictus*, *Ae. aegypti* and mosquitoes from the *Cx. pipiens* complex. These bacteria can be cultured and genetically manipulated for subsequent reinsertion into the insect host and after blood feeding increase its population size exponentially. In addition, transmission from males to females during mating can be exploited to colonize mosquitoes in nature [51, 52, 68].

Straif et al. [69] investigated the prevalence of bacteria in the midguts of malaria vectors and identified bacterial candidates for paratransgenesis in cultured and uncultured midgut bacteria from wild-caught *An. gambiae* and *An. funestus* mosquitoes. Twenty different genera of bacteria were identified in both mosquito samples, the most common bacteria being *Pantoea agglomerans*. Dong et al. [70] showed that when *Chryseobacterium meningosepticum* was introduced into *An. gambiae*, it became the dominant bacteria in the mosquito’s midgut, indicating that this species has a competitive advantage in the gut environment.

Although a variety of symbiotic bacteria have been identified in mosquito microflora, existing conventional culture techniques do not allow all the components of the microbiota inhabiting any niche or environmental sample to be isolated and identified because it is not possible to simulate the conditions required for their growth in a laboratory [16]. However, this drawback can be overcome by ribosomal RNA (rRNA)-based sequence studies [14].

Paratransgenic approaches can also work with fungal species, which have the advantage of surviving in the environment for months as spores and, unlike bacteria which need to be ingested to infect mosquitoes, can infect mosquitoes directly through the cuticle [71]. Fang et al. [72] genetically transformed *Metarhizium anisopliae* to express the effector molecule SM1 and the antimicrobial toxin scorpine, which interfere with *Plasmodium falciparum* development, reducing mosquito infectivity. There were also shown the presence of fungi in *Cx. quinquefasciatus* by Pidiyar et al. [14], who found *Aspergillus* and *Streptomyces spp*. in this mosquito. GM bacteria or fungi could potentially be used in paratransgenesis to produce an environment-friendly, highly effective specific biopesticide Thomas & Read [71].

#### Wolbachia

Rickettsiales, such as *Wolbachia*, an obligate intracellular gram-negative bacteria, can be found in the cytoplasmic vacuoles of insects, isopods, mites and nematodes [73, 74]. Maternally inherited, they can infect mosquito gonads and are therefore potential targets for delivering effector molecules in paratransgenic systems [53].

While many mosquito species can be infected by *Wolbachia*, epidemiologically important mosquitoes such as *Ae. albopictus* and *Cx.pipiens* are naturally infected, and the prevalence of *Wolbachia* in wild populations is high. There are several different phylogenetic groups of *Wolbachia*, and depending on the strain with which the mosquito is infected, crosses can be cytoplasmically compatible, incompatible or compatible in only one direction [75]. *Wolbachia* inflicts a severe selective pressure that quickly drives transovarial transmission of the bacteria through mosquito populations [76, 77].

There are three basic strategies for using *Wolbachia* to control vector populations: inserting the transgene directly into the *Wolbachia* genome and using cytoplasmic incompatibility mechanisms to suppress the target population and achieve the desired frequency in the target population; fixing the transgene in other cytoplasmic elements that are co-inherited with *Wolbachia* bacteria in the mosquito; and transforming the genome of the target mosquitoes and using cytoplasmic incompatibility as a gene insertion mechanism in the target population [74].

Transovarian transmission of *Wolbachia* has provoked considerable interest in paratransgenic systems, in which it is highly desirable to use bacteria that can spread through a population and are readily available [78, 79]. It is possible to infect several mosquito species with *Wolbachia* and produce different outcomes [80]. As *wPip Wolbachia* strains are found in the *Cx. pipiens* complex, exogenous bacteria do not need to be used, and control strategies can be based on *Wolbachia*. The transmission capacity and cytoplasmic incompatibility of this bacteria confirm its great potential for use in paratransgenesis. However, before it can be used, certain issues must be addressed. Firstly, intracellular bacteria require a cell culture to be maintained in the laboratory and, secondly, as effector molecules are excreted directly into the cytoplasm of host cells by *Wolbachia*, there must be a mechanism for these molecules to reach the hemolymph if they are to have the desired effect [45, 79, 81, 82].

Infection of *Ae. aegypti* by different *Wolbachia* strains leads to three outcomes: shortened lifespan, reducing their potential to transmit pathogens that have to infect adult mosquitoes to complete their development cycle [79]; limited susceptibility to infection with the dengue or chikungunya virus or the *Plasmodium* parasite [83]; and, depending on the strain of *Wolbachia*, induction of cytoplasmic incompatibility, with apparently no significant fitness cost and high horizontal transmission [79].

*An. gambiae* mosquitoes were found naturally infected with *Wolbachia* in wild population captured in Burkina Faso, Africa [84]. Hughes et al. [85] demonstrate that *An. gambiae* and *An.stephensi* microbiota prevent *Wolbachia* infection, but when the mosquito microbiota was removed with the use of antibiotics, *Wolbachia* was capable of infecting the mosquito and reaching maternal transmission to the progeny.

*Wolbachia* maternal transmission and further cytoplasmic incompatibility was successfully obtained by Bian et al. [86] in *An. stephensi* laboratory populations indicating that this bacteria has the potential to be used in a paratransgenic system for this species.

Although *Wolbachia* would appear to be an excellent candidate for a paratransgenesis system, caution should be exercised when using it, as Dodson et al. [87] showed that WNV infection was enhanced in *Wolbachia*-infected *Culex tarsalis*, a feature of *Wolbachia* that can jeopardize a control strategy based on this bacteria.

### Effector molecules

Richins et al. [88] developed a system that can be used to transport large molecules produced inside a bacterium and display them on its surface. The system has been successfully used to display enzymes and antibodies [45] and represents a major step forward in the search for a successful paratransgenic system.

Testing for effector molecules for use in paratransgenesis systems, Riehle et al. [46] showed that the formation of *Plasmodium berghei* oocysts in *An. stephensi* mosquitoes can be reduced by expression of SM1 and mPLA2 by commensal recombinant *Escherichia coli*.

Yoshida et al. [53] were able to completely block the development of *P. berghei* in the midgut of *An. stephensi* mosquitoes using a single-chain antibody fragment (scFv) excreted by recombinant *E. coli*. However, these bacteria cannot survive in mosquitoes and disappears quickly from their midguts, making it unsuitable for use in the field [46]. For bacteria to be used in paratransgenesis programs in the field they must not only be adapted to the digestive tract of mosquitoes, but also be able to compete with other bacteria in the digestive tract as well as have a lifetime compatible with the adult mosquito vector so they can readily colonize the midgut [46].

There are several bacteria and fungi that can be transformed to express effector molecules for use in a paratransgenic system. These include *M. anisopliae*, a fungus which can colonize *Ae. aegypti* mosquitoes [89, 90], and be genetically modified to express scorpine, a molecule that can be used to interfere with dengue [72, 91]. Monoclonal antibodies can be used as effector molecules both with *Pantoea agglomerans* or *Sodalis glossinidius*, to form an antipathogenic system [92–94]. Different combinations of anti-*Plasmodium* effector molecules (cecropin A, SM1, Scorpine, EPIP, scFvs and mPLA2) secreted by transgenic *Pa. agglomerans* inhibited the development of the human malaria parasite *P. falciparum* and the rodent malaria parasite *P. berghei* in *An. gambiae* and *An. stephensi* mosquitoes [66, 95].

### Risk assessment

As with any GM organism, a number of key points must be addressed before a paratransgenesis system can be utilized in the field. Indeed, one of the most challenging aspects of risk assessment for the use of GM organism is the identification of potential problems. However, most of the harmful effects are known and include adverse effects on biological diversity, adverse consequences for gene flow and environmental changes [11].

Cost-benefit analyses are required to provide justification for new types of intervention. However, the lack of reliable data on the monetary impact of neglected tropical diseases such as dengue means that it is unclear whether the costs of developing and implementing these forms of intervention are justifiable or not. Ideally, it should be possible not only to analyze the cost-effectiveness of new strategies, but also to compare them with available alternatives and model their incorporation into integrated-vector management programs that include drugs and vaccines when these are available [11].

Although safety guidelines for field use of paratransgenic mosquitoes are strict, most of the problems associated with releasing GMM are not present in the paratransgenic approach, which is compatible with traditional control strategies and IPM programs.

The risk associated with the possibility that the modified bacteria could also infect “non-target” insect species is drastically reduced due to the fact that males will seek females from the same species. When a paratransgenesis system is designed as a population suppression strategy (Self-Limiting) there will be a selective pressure that inevitably drives the lethal gene to extinction and even if the chosen strategy is to create an etiological agent refractory mosquito (Self-sustaining), without a gene driver the transgene is bound to fade and disappear [11]. Another aspect to be considered is that there is no selective pressure interacting with the midgut bacteria as the mosquito is a dead end host [45].

Nonetheless, any GM organism release project shall undergo an environmental risk assessment to evaluate potential adverse effects on human and animal health. The European Food Safety Authority (EFSA) is a regulatory agency that regulates, identifies and appraises environmental risks as well as potential adverse effects of the GM organisms released on the environment. The regulatory process guidelines are as follows: (a) identify and characterize potential adverse effects caused by the GM organism; (b) assess potential consequences of each adverse effect; (c) evaluate the likelihood of each potential adverse effect; (d) each GM organisms characteristics should be analyzed for risk estimation; (e) plan for risk management strategies for release and marketing of GM organisms; (f) define the overall risk of the releasing of GM organisms [96].

The risk assessment should preferably result in quantitative data, however qualitative data is acceptable in specific scenarios. Assumptions made must be clarified and all circumstances discussed regarding its uncertainties associated with the identified risks. The acceptable evaluation outcome of releasing GM organisms should mandatorily imply risk levels far lower than its benefits [96].

## Conclusions

Previous studies have found multiple paratransgenesis candidates in various mosquito species. Each type of bacteria has certain advantages and disadvantages, and the choice of which to use is governed by the particular vector-control approach being used. The advantage of using GM symbionts rather than transgenic mosquitoes is that the former is a relatively simple approach and, unlike the genetic transformation of mosquitoes, does not have the disadvantage of potentially affecting mosquito fitness. In addition, the close association between symbionts and their hosts provides a method for the transgene to spread quickly through the target population [97].

One of the most problematic issues of strategies based on GMM is that a transgenic mosquito strain is required for every sibling species. Even if an effective drive mechanism is found, the number of sibling species must be considered. For example, *An. gambiae*, the most important vector of malaria in Africa, has many reproductively isolated strains, greatly limiting the effectiveness of GMM [14, 98, 99]. A paratransgenic system can use the same GM bacteria for different mosquito species and even different genera, the only requirement being that the bacteria used be able to survive and colonize the mosquito host in sufficient numbers so that effector molecule expression reaches the desired level, thus overcoming the problem highlighted above and increasing the range of mosquitoes that can be controlled [14, 45, 46, 64].

Genetic transformation of mosquitoes is far more difficult and complex than paratransgenesis, in which effector molecules can be expressed and excreted by simple bacterial systems [53]. The successful transformation of *P. agglomerans* has substantially improved the expression of effector molecules and proteins in the mosquito midgut because of its capacity to rapidly replicate after a blood meal [66]. If the GM bacteria successfully express the effector molecule but fail to excrete it or display it on their surface, an apoptotic mechanism must be used, leading to extra fitness costs. However, if the effector molecules are excreted by the bacteria they can be produced continually, significantly increasing the effectiveness of the paratransgenic system. Fortunately, there are mechanisms such as the outer membrane protein A (OmpA) that allow bacteria to export molecules to the environment [45, 88].

Paratransgenic strategies may prove very useful for controlling mosquito species such as *Culex* that are, for as yet unknown reasons, inherently difficult to transform and have been genetically modified only once by the embryo microinjection technique [100, 101]. In contrast, many other mosquito species have been successfully transformed more than once (*Ae. aegypti*, *Ae. albopictus*, *Aedes fluviatilis*, *An. gambiae* and *An. stephensi*) [102–105].

Microorganisms associated with vectors can exert pathogenic effects on the host, interfere with its reproduction and reduce its vector competence [24, 52]. Paratransgenesis may prove to be a highly valuable tool for use in IPM and is potentially of particular value in areas where there are species complexes or more than one species whose population needs to be reduced or made refractory to a given pathogen, as well as in the control of mosquito species that are not easily transformed.

## References

[1] WHO Global Malaria Programme (2012). New report signals slowdown in the fight against malaria 2012.

[2] WHO-TDR. Scientific Working Group Report on Dengue. 2006.

[3] World Health Organization (2012). Global Strategy for dengue prevention and control, 2012–2020: WHO Report.

[4] WHO - Progress report 2000–2009 and strategic plan 2010–2020 of the global programme to eliminate lymphatic filariasis: halfway towards eliminating lymphatic filariasis. 2010 World Health Organization. ISBN 978-92-4-150072-2

[5] Dorta DM, Vasuki V, Rajavel A (1993). Evaluation of organophosphorus and synthetic pyrethroid insecticides against six vector mosquitoe species. Rev Saude Publica.

[6] Gubler DJ (2003). The changing epidemiology of yellow fever and dengue, 1900 to 2003: Full circle?. Comp Immunol Microb Infect Dis.

[7] Nicholson GM (2007). Fighting the global pest problem: preface to the special toxicon issue on insecticidal toxins and their potential for insect pest control. Toxicon.

[8] Reiter P (2007). Oviposition, dispersal and survival in *Aedes aegypti*: Implications for the efficacy of control strategies. Vector-Borne Zoonot.

[9] Fonseca DM, Smith JL, Wilkerson RC, Fleischer RC (2006). Pathways of expansion and multipleintroductionsillustrated by largegeneticdifferentiation among worldwidepopulations of the southernhousemosquito. Am J Trop Med Hyg.

[10] Breman JG, Egan A, Keusch GT (2001). The intolerable burden of malaria: a new look at the numbers. Am J Trop Med Hyg.

[11] WHO - TDR. Planning meetings on Progress and Prospects for the Use of Genetically Modified Mosquitoes to Prevent Disease Transmission: Meeting 1. Technical Consultations on the Current Status and Planning for Future Development. 2009.

[12] Mairiang D, Zhang H, Sodja A, Murali T, Suriyaphol P, Malasit P, Limjindaporn T, Finley RL (2013). Identification of New Protein Interactions between DengueFever Virus and Its Hosts, Human and Mosquito. PLoS One.

[13] Forattini OP (2002). Culicidologia médica.

[14] Pidiyar VJ, Jangid K, Patole MS, Shouche YS (2004). Studies on cultured and uncultured microbiota of wild *Culex quinquefasciatus* mosquito midgut based on 16s ribosomal RNA gene analysis. Am J Trop Med Hyg.

[15] Fontes G, Brito AC, Calheiros CML, Antunes CMF, Rocha EMM (1994). Situação Atual da Filariose Bancroftiana na Cidade de Maceió, Estado de Alagoas, Brasil. Cad Saúde Públ.

[16] Huhn GD, Sejvar JJ, Montgomery SP, Dworkin MS (2003). West Nile Virus in the United States: an update on an emerging infectious disease. Am Fam Physician.

[17] Knipling E (1955). Possibilities of insect control or eradication through use of sexually sterile males. J Econ Entomol.

[18] Besansky NJ, Collins FH (1992). The mosquito genome: organization, evolution and manipulation. Parasitol Today.

[19] Mackenzie JS, Gubler DJ, Petersen LR (2004). Emerging flaviviruses: The spread and resurgence of Japanese encephalitis, West Nile and dengue virus. Nat Med.

[20] Pates H, Curtis CF (2005). Mosquito behavior and vector control. Annu Rev Entomol.

[21] Vreysen M, Robinson AS, Hendrichs J (2007). Area-Wide Control of Insect Pests: From Research to Field Implementation.

[22] Axtell RC, Arends JJ (1990). Ecology and Management of Arthropod Pests of Poultry. Annu Rev Entomol.

[23] Bracco JE, Dalbon M, Marinotti O, Barata JM (1997). Resistance to organophosphorous and carbamates insecticides in a population of *Culex quinquefasciatus*. Rev Saude Publica.

[24] Pocquet N, Milesi P, Makoundou P, Unal S, Zumbo B, Atyame C, Darriet F, Dehecq JS, Thiria J, Bheecarry A, Iyaloo DP, Weill M, Chandre F, Labbé P (2013). Multiple insecticide resistances in the disease vector Culex p. quinquefasciatus from Western Indian Ocean. PLoS One.

[25] Govindarajan M, Sivakumar R (2014). Larvicidal, ovicidal, and adulticidal efficacy of Erythrina indica (Lam.) (Family: Fabaceae) against *Anopheles stephensi*, *Aedes aegypti*, and *Culex quinquefasciatus* (Diptera: Culicidae). Parasitol Res.

[26] Taipe-Lagos CB, Natal D (2003). Abundância de culicídeos em área metropolitana Preservada e suas Implicações Epidemiológicas. Rev Saude Publica.

[27] Natal D, Araújo FAA, Vianna RST, Pereira LE, Ueno HM (2004). O mosquito das águas poluídas.

[28] Cardoso JC, Corseuil E, Barata JMS (2005). Culicinae (Diptera, Culicidae) ocorrentes no Estado do Rio Grande do Sul, Brasil. Rev Bras Entomol.

[29] Curtis CF (1968). A possible genetic method for the control of insect pests, with special reference to tsetse flies. Bull Entomol Res.

[30] Jasinskiene N, Coleman J, Ashikyan A, Salampessy M, Marinotti O, James AA (2007). Genetic control of malaria parasite transmission: threshold levels for infection in an avian model system. Am J Trop Med Hyg.

[31] Wilke AB, Marrelli MT (2012). Genetic control of mosquitoes: populationsuppressionstrategies. Rev Inst Med Trop Sao Paulo.

[32] Moreira LA, Edwards MJ, Adhami F, Jasinskiene N, James AA, Jacobs-Lorena M (2000). Robustgut-specificgeneexpression in transgenic *Aedesaegypti* mosquitoes. Proc Natl Acad Sci U S A.

[33] Ito J, Ghosh A, Moreira LA, Wimmer EA, Jacobs-Lorena M (2002). Transgenic anopheline mosquitoes impaired in transmission of a malaria parasite. Nature.

[34] Kim W, Koo H, Richman AM, Seeley D, Vizioli J, Klocko AD, O'Brochta DA (2004). Ectopic expression of a cecropin transgene in the human malaria vector mosquito *Anopheles gambiae* (Diptera: Culicidae): effects on susceptibility to *Plasmodium*. J Med Entomol.

[35] Franz AW, Sanchez-Vargas I, Adelman ZN, Blair CD, Beaty BJ, James AA, Olson KE (2006). Engineering RNA interference-based resistance to dengue virus type 2 in genetically modified *Aedes aegypti*. Proc Natl Acad Sci U S A.

[36] Moreira LA, Ito J, Ghosh A, Devenport M, Zieler H, Abrahan EG, Crisanti A, Nolan T, Catteruccia F, Jacobs-Lorena M (2002). Bee venom phospholipase Inhibits malaria parasite development in transgenic mosquitoes. J Biol Chem.

[37] Isaacs AT, Li F, Jasinskiene N, Chen X, Nirmala X, Marinotti O, Vinetz JM, James AA (2011). Engineered resistance to *Plasmodium falciparum* development in transgenic *Anopheles stephensi*. PLoS Pathog.

[38] Meredith JM, Basu S, Nimmo DD, Larget-Thiery I, Warr EL, Underhill A, McArthur CC, Carter V, Hurd H, Bourgouin C, Eggleston P (2011). Site-specific integration and expression of an anti-malarial gene in transgenic *Anopheles gambiae* significantly reduces *Plasmodium* infections. PLoS One.

[39] Benedict MQ, Robinson AS (2003). The first releases of transgenic mosquitoes: an argument for the sterile insect technique. Trends Parasitol.

[40] Heinrich JC, Scott MJ (2000). A repressible female-specific lethal genetic system for making transgenic insect suitable for a sterile-release program. Proc Natl Acad Sci U S A.

[41] Thomas DD, Donnelly CA, Wood RJ, Alphey L (2000). Insect Population Control Using a Dominant, Repressible, Lethal genetic Sistem. Science.

[42] Coleman PG, Alphey L (2004). Genetic control of vector populations: an imminent prospect. Trop Med Int Health.

[43] Fortini M, Simon M, Rubin G (1992). Signalling by the sevenless protein tyrosine kinase is mimicked by Ras1 activation. Nature.

[44] Alphey L (2002). Re-engineering the sterile insect technique. Insect Biochem Mol Biol.

[45] Riehle MA, Jacobs-Lorena M (2005). Using bacteria to express and display anti-parasite molecules in mosquitoes: current and future strategies. Insect Biochem Mol Biol.

[46] Riehle MA, Moreira CK, Lampe D, Lauzon C, Jacobs-Lorena M (2007). Using bacteria to express and display anti-*Plasmodium* molecules in the mosquito midgut. Int J Parasitol.

[47] Beard CB, Mason PW, Aksoy S, Tesh RB, Richards FF (1992). Transformation of an insect symbiont and expression of a foreign gene in the Chagas disease vector *Rhodnius prolixus*. Am J Trop Med Hyg.

[48] Beard CB, O’Neill SL, Tesh RB, Richards FF, Aksoy S (1993). Modification of arthropod vector competence via symbiotic bacteria. Parasitol Today.

[49] Chavshin AR, Oshaghi MA, Vatandoost H, Pourmand MR, Raeisi A, Enayati AA, Mardani N, Ghoorchian S (2012). Identification of bacterial microflora in the midgut of the larvae and adult of wild caught *Anopheles stephensi*: a step toward finding suitable paratransgenesis candidates. Acta Trop.

[50] Conte JE (1997). A novel approach to preventing insect-borne diseases. N Engl J Med.

[51] Beard CB, Cordon-Rosales C, Durvasula RV (2002). Bacterial symbionts of the triatominae and their potential use in control of Chagas disease transmission. Annu Rev Entomol.

[52] Favia G, Ricci I, Damiani C, Raddadi N, Crotti E, Marzorati M, Rizzi A, Urso R, Brusetti L, Borin S, Mora D, Scuppa P, Pasqualini L, Clementi E, Genchi M, Corona S, Negri I, Grandi G, Alma A, Kramer L, Esposito F, Bandi C, Sacchi L, Daffonchio D (2007). Bacteria of the genus *Asaia*stably associate with *Anopheles stephensi*, an Asian malarial mosquito vector. Proc Natl Acad Sci U S A.

[53] Yoshida S, Ioka D, Matsuoka H, Endo H, Ishii A (2001). Bacteria expressing single-chain immunotoxin inhibit malaria parasite development in mosquitoes. Mol Biochem Parasitol.

[54] Aksoy S, Weiss B, Attardo G (2008). Paratransgenesis applied for control of tsetse transmitted sleeping sickness. Adv Exp Med Biol.

[55] Coutinho-Abreu IV, Zhu KY, Ramalho-Ortigao M (2009). Transgenesis and paratransgenesis to control insect-borne diseases: current status and future challenges. ParasitolInt.

[56] Pumpuni CB, Demaio J, Kent M, Davis JR, Beier JC (1996). Bacterial population dynamics in three anopheline species: the impact on *Plasmodium* sporogonic development. Am J Trop Med Hyg.

[57] Gonzalez-Ceron L, Santillan F, Rodriguez MH, Mendez D, Hernandez-Avila JE (2003). Bacteria in midguts of field-collected *Anopheles albimanus* block *Plasmodium vivax* sporogonic development. J Med Entomol.

[58] Lindh JM, Terenius O, Faye I (2005). 16S rRNA gene-based identification of midgut bacteria from field-caught *Anopheles gambiae* sensu lato and *A. funestus* mosquitoes reveals new species related to known insect symbionts. Appl Environ Microbiol.

[59] Damiani C, Ricci I, Crotti E, Rossi P, Rizzi A, Scuppa P, Esposito F, Bandi C, Daffonchio D, Favia G (2008). Paternal transmission of symbiotic bacteria in malaria vectors. Curr Biol.

[60] Terenius O, de Oliveira CD, Pinheiro WD, Tadei WP, James AA, Marinotti O (2008). 16S rRNA gene sequences from bacteria associated with adult *Anopheles darlingi* (Diptera: Culicidae) mosquitoes. J Med Entomol.

[61] Rani A, Sharma A, Rajagopal R, Adak T, Bhatnagar RK (2009). Bacterial diversity analysis of larvae and adult midgut microflora using culture-dependent and culture-independent methods in lab-reared and field-collected *Anopheles stephensi*-an Asian malarial vector. BMC Microbiol.

[62] Hillesland H, Read A, Subhadra B, Hurwitz I, McKelvey R, Ghosh K, Das P, Durvasula R (2008). Identification of aerobic gut bacteria from the kala azar vector, *Phlebotomus argentipes*: a platform for potential paratransgenic manipulation of sand flies. Am J Trop Med Hyg.

[63] Gaio AO, Gusmão DS, Santos AV, Berbert-Molina MA, Pimenta PF, Lemos FJ (2011). Contribution of midgut bacteria to blood digestion and egg production in *Aedes aegypti* (Diptera: culicidae). Parasit Vectors.

[64] Sayler GS, Ripp S (2000). Field applications of genetically engineered microorganisms for bioremediation processes. Curr Opin Biotechnol.

[65] Briones AM, Shililu J, Githure J, Novak R, Raskin L (2008). *Thorsellia anophelis* is the dominant bacterium in a Kenyan population of adult *Anopheles gambiae* mosquitoes. ISME J.

[66] Wang S, Ghosh AK, Bongio N, Stebbings KA, Lampe DJ, Jacobs-Lorena M (2012). Fighting malaria with engineered symbiotic bacteria from vector mosquitoes. Proc Natl Acad Sci U S A.

[67] Dinparast DN, Jazayeri H, Raz A, Favia G, Ricci I, Zakeri S (2011). Identification of the midgut microbiota of An. stephensi and An. maculipennis for their application as a paratransgenic tool against malaria. PLoS One.

[68] De Freece C, Damiani C, Valzano M, D'Amelio S, Cappelli A, Ricci I, Favia G (2014). Detection and isolation of the α-proteobacterium *Asaia* in *Culex* mosquitoes. Med Vet Entomol.

[69] Straif SC, Mbogo CN, Toure AM, Walker ED, Kaufman M, Toure YT, Beier JC (1998). Midgut bacteria in *Anopheles gambiae* and *An. funestus* (Diptera: Culicidae) from Kenya and Mali. J Med Entomol.

[70] Dong Y, Manfredini F, Dimopoulos G (2009). Implication of the mosquito midgut microbiota in the defense against malaria parasites. PLoS Pathog.

[71] Thomas MB, Read AF (2007). Can fungal biopesticides control malaria?. Nat Rev Microbiol.

[72] Fang W, Vega-Rodríguez J, Ghosh AK, Jacobs-Lorena M, Kang A, St Leger RJ (2011). Development of transgenic fungi that kill human malaria parasites in mosquitoes. Science.

[73] Laven H (1967). Eradication of *Culex pipiens fatigans* through cytoplasmic incompatibility. Nature.

[74] Townson H (2002). *Wolbachia* as a potentialtool for suppressing filarial transmission. Ann Trop Med Parasitol.

[75] Atyame CM, Pasteur N, Dumas E, Tortosa P, Tantely ML, Pocquet N, Licciardi S, Bheecarry A, Zumbo B, Weill M, Duron O (2011). Cytoplasmic incompatibility as a means of controlling *Culex pipiens quinquefasciatus* mosquito in the islands of the south-western Indian Ocean. PLoS Negl Trop Dis.

[76] Werren JH, Baldo L, Clark ME (2008). *Wolbachia*: master manipulators of invertebrate biology. Nat Rev Microbiol.

[77] Calvitti M, Moretti R, Skidmore AR, Dobson SL (2012). *Wolbachia* strain *wPip* yields a pattern of cytoplasmic incompatibility enhancing a *Wolbachia*-based suppression strategy against the disease vector *Aedes albopictus*. Parasit Vectors.

[78] Walker T, Johnson PH, Moreira LA, Iturbe-Ormaetxe I, Frentiu FD, McMeniman CJ, Leong YS, Dong Y, Axford J, Kriesner P, Lloyd AL, Ritchie SA, O'Neill SL, Hoffmann AA (2011). The *wMel Wolbachia* strain blocks dengue and invades caged *Aedes aegypti* populations. Nature.

[79] Weiss BL, Mouchotte R, Rio RV, Wu YN, Wu Z, Heddi A, Aksoy S (2006). Interspecific transfer of bacterial endosymbionts between tsetse fly species: infection establishment and effect on host fitness. Appl Environ Microbiol.

[80] Zimmer C (2001). *Wolbachia*, *a* tale of sex and survival. Science.

[81] Dobson SL, Bourtzis K, Braig HR, Jones BF, Zhou W, Rousset F, O'Neill SL (1999). *Wolbachia* infections are distributed throughout insect somatic and germ line tissues. Insect Biochem Mol Biol.

[82] Moreira LA, Iturbe-Ormaetxe I, Jeffery JA, Lu G, Pyke AT, Hedges LM, Rocha BC, Hall-Mendelin S, Day A, Riegler M, Hugo LE, Johnson KN, Kay BH, McGraw EA, van den Hurk AF, Ryan PA, O'Neill SL (2009). A *Wolbachia* symbiont in *Aedes aegypti* limits infection with dengue, Chikungunya, and *Plasmodium*. Cell.

[83] Baldini F, Segata N, Pompon J, Marcenac P, Robert Shaw W, Dabiré RK, Diabaté A, Levashina EA, Catteruccia F (2014). Evidence of natural *Wolbachia* infections in field populations of *Anopheles gambiae*. Nat Commun.

[84] Hughes GL, Dodson BL, Johnson RM, Murdock CC, Tsujimoto H, Suzuki Y, Patt AA, Cui L, Nossa CW, Barry RM, Sakamoto JM, Hornett EA, Rasgon JL (2014). Native microbiome impedes vertical transmission of *Wolbachia* in Anopheles mosquitoes. Proc Natl Acad Sci U S A.

[85] Bian G, Joshi D, Dong Y, Lu P, Zhou G, Pan X, Xu Y, Dimopoulos G, Xi Z (2013). *Wolbachia* invades *Anopheles stephensi* populations and induces refractoriness to *Plasmodium* infection. Science.

[86] Dodson BL, Hughes GL, Paul O, Matacchiero AC, Kramer LD, Rasgon JL (2014). *Wolbachia* enhances West Nile virus (WNV) infection in the mosquito *Culex tarsalis*. PLoS Negl Trop Dis.

[87] Richins RD, Kaneva I, Mulchandani A, Chen W (1997). Biodegradation of organophosphorus pesticides by surface-expressed organophosphorus hydrolase. Nat Biotechnol.

[88] Paula AR, Carolino AT, Silva CP, Samuels RI (2011). Susceptibility of adult females *Aedes aegypti* (Diptera: Culicidae) to the entomopathogenic fungus *Metarhizium anisopliae* is modified following blood feeding. Parasit Vectors.

[89] Paula AR, Carolino AT, Paula CO, Samuels RI (2011). The combination of the entomopathogenic fungus *Metarhizium anisopliae* with the insecticide Imidacloprid increases virulence against the dengue vector *Aedes aegypti* (Diptera: Culicidae). Parasit Vectors.

[90] Carballar-Lejarazú R, Rodríguez MH, de la Cruz Hernández-Hernández F, Ramos-Castañeda J, Possani LD, Zurita-Ortega M, Reynaud-Garza E, Hernández-Rivas R, Loukeris T, Lycett G, Lanz-Mendoza H (2008). Recombinant scorpine: a multifunctional antimicrobial peptide with activity against different pathogens. Cell Mol Life Sci.

[91] Durvasula RV, Gumbs A, Panackal A, Kruglov O, Taneja J, Kang AS, Cordon-Rosales C, Richards FF, Whitham RG, Beard CB (1999). Expression of a functional antibody fragment in the gut of *Rhodnius prolixus* via transgenic bacterial symbiont *Rhodococcus rhodnii*. Med Vet Entomol.

[92] Bisi DC, Lampe DJ (2011). Secretion of anti-Plasmodium effector proteins from a natural *Pantoea agglomerans* isolate by using PelB and HlyA secretion signals. Appl Environ Microbiol.

[93] De Vooght L, Caljon G, Stijlemans B, De Baetselier P, Coosemans M, Van den Abbeele J (2012). Expression and extracellular release of a functional anti-trypanosome Nanobody® in *Sodalis glossinidius*, a bacterial symbiont of the tsetse fly. Microb Cell Fact.

[94] Isaacs AT, Jasinskiene N, Tretiakov M, Thiery I, Zettor A, Bourgouin C, James AA (2012). Transgenic *Anopheles stephensi* coexpressing single-chain antibodies resist *Plasmodium falciparum* development. Proc Natl Acad Sci U S A.

[95] EFSA (2013). Panel on Genetically Modified Organisms (GMO) - Guidance on the environmental risk assessment of genetically modified animals.

[96] Durvasula RV, Gumbs A, Panackal A, Kruglov O, Aksoy S, Merrifield RB, Richards FF, Beard CB (1997). Prevention of insect-borne disease: an approach using transgenic symbiotic bacteria. Proc Natl Acad Sci U S A.

[97] Della Torre A, Costantini C, Besansky NJ, Caccone A, Petrarca V, Powell JR, Coluzzi M (2002). Speciation within *Anopheles gambiae*--the glass is half full. Science.

[98] Coetzee M (2004). Distribution of the African malaria vectors of the *Anopheles gambiae* complex. Am J Trop Med Hyg.

[99] Allen ML, O'Brochta DA, Atkinson PW, Levesque CS (2001). Stable, germ-line transformation of *Culexquinquefasciatus* (Diptera: Culicidae). J Med Entomol.

[100] Allen ML, Christense BM (2004). Flight muscle-specific expression of *act88F*: GFP in transgenic *Culex quinquefasciatus* Say (Diptera: culicidae). Parasitol Int.

[101] Miller LH, Sakai RK, Romans P, Gwadz RW, Kantoff P, Coon HG (1987). Stable integration and expression of a bacterial gene in the mosquito *Anopheles gambiae*. Science.

[102] Jasinskiene N, Coates CJ, Benedict MQ, Cornel AJ, Rafferty CS, James AA, Collins FH (1998). Stable transformation of the yellow fever mosquito, *Aedesaegypti*, with the Hermes element from the housefly. Proc Natl Acad Sci U S A.

[103] Catteruccia F, Nolan T, Loukeris TG, Blass C, Savakis C, Kafatos FC, Crisanti A (2000). Stable germline transformation of the malaria mosquito *Anophelesstephensi*. Nature.

[104] Labbé GM, Nimmo DD, Alphey L (2010). Piggybac and PhiC31 mediated genetic transformation of the Asian tiger mosquito, *Aedesalbopictus*(Skuse). PLoS Negl Trop Dis.

[105] Rodrigues FG, Oliveira SB, Rocha BC, Moreira LA (2006). Germline transformation of *Aedesfluviatilis* (Diptera:Culicidae) with the piggyBac transposable element. Mem Inst Oswaldo Cruz.

